# P03-03 The impact of regular physical education, physical activity and sport provision in second level schools on adolescent physical activity behaviours: A systematic literature review

**DOI:** 10.1093/eurpub/ckac095.039

**Published:** 2022-08-29

**Authors:** Padraic Rocliffe, Liam Walsh, Ciaran MacDonncha, Patricia Mannix-McNamara, Brendan O' Keeffe

**Affiliations:** 1 Physical Education and Sport Sciences, University of Limerick, Limerick, Ireland; 2 School of Education, University of Limerick, Limerick, Ireland

**Keywords:** Physical education, sport, school, physical activity behaviour, systematic literature review

## Abstract

**Background:**

Regular engagement in physical activity (PA) is cited as a powerful predicter of future health among adolescents. International guidelines recommend sixty-minutes of moderate-to-vigorous-intensity PA daily for adolescents. Prevalence of physical inactivity is high among adolescents and is regarded as a leading risk factor for death worldwide, contributing to the onset of non-communicable diseases with just 20% of adolescents meeting the recommended PA guidelines. Physical inactivity cost $67.5 billion worldwide in 2013 and is estimated to reach over? 110 billion in 2030. Physical Education (PE) is recognized as playing an integral role in the promotion of PA and health. Despite the worldwide adoption of school PE, PA and sport policies to promote PA and health, paralleled with significant investment, a gap in the literature exists that synthesises the impact of school PE, PA and sport on adolescent PA behaviours

**Methods:**

Web of Science, SPORTDiscus, PsychINFO, ERIC and MEDLINE were searched for articles that examined the impact of regular school PE, PA and sport provision on adolescent (12-18 years) published between 2000-2020.

**Results:**

Preliminary results indicate n = 39 articles have met the inclusion criteria. Gender differences are observed on the impact of PE on daily Moderate to Vigorous Physical Activity (MVPA) and overall daily PA. Studies that examined the impact of PE, PA and sport on adolescent PA behaviours outside of school are less frequent indicating a paucity of evidence in this research area.

**Conclusion:**

Such research is essential to synthesise a deeper understanding on whether current provision is impactful and/or requiring modification and thus will provide comprehensive evidence for 1) sustained financial investment; 2) modification of existing provision to potentiate positive impact and 3) reducing the international cost of physical inactivity. The central premise of this systematic literature review is that current provision is impactful and of enormous benefit.

